# The mobile-phone-based iCO^TM^ Smokerlyzer^®^: Comparison with the piCO^+^ Smokerlyzer^®^ among smokers undergoing methadone-maintained therapy

**DOI:** 10.18332/tid/111355

**Published:** 2019-09-09

**Authors:** Hsui Yang Wong, Muniswary Subramaniyan, Chris Bullen, A. N. Amer Siddiq, Mahmoud Danaee, Anne Yee

**Affiliations:** 1 Department of Psychological Medicine, University of Malaya Center of Addiction Sciences (UMCAS), Kuala Lumpur, Malaysia; 2 National Institute for Health Innovation, The University of Auckland, Auckland, New Zealand; 3 Department of Social and Preventive Medicine, University of Malaya, Kuala Lumpur, Malaysia

**Keywords:** nicotine, cessation, nicotine dependence, smoking/harm reduction

## Abstract

**INTRODUCTION:**

The mobile-phone-based Bedfont iCO^TM^ Smokerlyzer^®^ is of unknown validity and reproducibility compared to the widely-used piCO^+^ Smokerlyzer^®^. We aimed to compare the validity and reproducibility of the iCO^TM^ Smokerlyzer^®^ with the piCO^+^ Smokerlyzer^®^ among patients reducing or quitting tobacco smoking.

**METHODS:**

Methadone-maintained therapy (MMT) users from three centers in Malaysia had their exhaled carbon monoxide (eCO) levels recorded via the piCO^+^ and iCO^TM^ Smokerlyzers^®^, their nicotine dependence assessed with the Malay version of the Fagerström Test for Nicotine Dependence (FTND-M), and daily tobacco intake measured via the Opiate Treatment Index (OTI) Tobacco Q-score. Pearson partial correlations were used to compare the eCO results of both devices, as well as the corresponding FTND-M scores.

**RESULTS:**

Among the 146 participants (mean age 47.9 years, 92.5% male, and 73.3% Malay ethnic group) most (55.5%) were moderate smokers (6-19 cigarettes/day). Mean eCO categories were significantly correlated between both devices (r=0.861, p<0.001), and the first and second readings were significantly correlated for each device (r=0.94 for the piCO^+^ Smokerlyzer^®^, p<0.001; r=0.91 for the iCO^TM^ Smokerlyzer^®^, p<0.001). Exhaled CO correlated positively with FTND-M scores for both devices. The post hoc analysis revealed a significantly lower iCO^TM^ Smokerlyzer^®^ reading of 0.82 (95% CI: 0.69–0.94, p<0.001) compared to that of the piCO^+^ Smokerlyzer^®^, and a significant intercept of -0.34 (95% CI: -0.61 – -0.07, p=0.016) on linear regression analysis, suggesting that there may be a calibration error in one or more of the iCO^TM^ Smokerlyzer^®^ devices.

**CONCLUSIONS:**

The iCO^TM^ Smokerlyzer^®^ readings are highly reproducible compared to those of the piCO^+^ Smokerlyzer^®^, but calibration guidelines are required for the mobile-phone-based device. Further research is required to assess interchangeability.

## INTRODUCTION

Objective smoking measures are useful to verify self-reported smoking status by individuals participating in harm reduction or smoking cessation interventions and studies^1^. Exhaled carbon monoxide (eCO) is widely used for these purposes because it is less expensive and less invasive than other measures, such as serum or urinary cotinine, and provides immediate results that assist in motivating patients to quit^1,2^. The piCO^+^ Smokerlyzer^®^ is commonly used to measure eCO levels in clinical and research settings^3^ because of its high validity and reproducibility in discriminating smokers from non-smokers^4-6^.

To date, the validity and reproducibility of the iCO^TM^ Smokerlyzer^®7^, an eCO measuring device designed for use with a smartphone and marketed primarily as a self-monitoring tool, is unknown. If found to perform as well as the piCO^+^ Smokerlyzer^®^, despite being intended for single-patient use, the iCO^TM^ Smokerlyzer^®^ could potentially be a research tool in smoking intervention studies, and at a lower cost. In clinical settings, the iCO^TM^ Smokerlyzer^®^ may also be useful where limited devices are available and where frequent monitoring may support therapeutic goals such as harm reduction for individuals on methadone-maintenance therapy (MMT)^8,9^.

This exploratory study aimed to compare the validity and reproducibility of the iCO^TM^ Smokerlyzer^®^ with those of the piCO^+^ Smokerlyzer^®^, and correlate eCO levels with an established measure of nicotine dependence.

## METHODS

### Study design

Participants from three methadone clinics (University Malaya Medical Center, San Peng, and Chow Kit) in Kuala Lumpur, Malaysia were recruited from December 2017 to January 2018. Participants who were aged ≥18 years, on MMT for two months or more, who had an established therapeutic compliance and were not on marijuana or any other recreational drugs (as determined by routine urinary drug tests), and smoked at least one cigarette daily for the past one month, were approached for consent to take part in the study. Participants who could not understand the device instructions or who were medically unstable were excluded. The Medical Ethical Committee of University Malaya Medical Center approved the study protocol (MECID: 20146-331).

### Procedure

Sociodemographic data were obtained and the Malay version of the Fagerström Test for Nicotine Dependence (FTND-M) was administered^10^. This instrument has moderate validity in distinguishing smokers with nicotine dependence from their non-nicotine-dependent counterparts with a cut-off of 2, and positively correlates with piCO^+^ Smokerlyzer^®-^measured eCO levels^10,11^.

The tobacco Q-score^12^ was calculated by dividing by two the total number of cigarettes consumed on the two days before the day of the study, enabling each participant to be categorized as light (≤5 cigarettes/ day), moderate (6–19 cigarettes/day) or heavy smoker (≥20 cigarettes/day)^13,14^.

Both devices were sanitized with anti-bacterial cleaning wipes between participants as per manufacturer recommendations^3,7^, using makeshift single-use mouthpieces for both devices to reduce transmission of fluids between participants; the smartphone Smokerlyzer application was used with the iCO^TM^ Smokerlyzer^®^. The instructions for both devices were: to completely exhale, take a deep breath, hold the breath for 15 s, and exhale completely and slowly into each device, which yielded values in parts per million (ppm). Each participant provided four samples with 5-minute intervals between samples, beginning with the piCO^+^ Smokerlyzer^®^, and alternating with the iCO^TM^ Smokerlyzer^®^.

### Data analysis

Descriptive statistics were used to examine characteristics data. The piCO^+^ Smokerlyzer^®^ readings were converted into ordinal categories (0–6; 7–10; 11–15; 16–20; 21–25; 26–30; and ≥31 ppm) to be compared with the ordinal categories in the initial results interface obtained from the iCO^TM^ Smokerlyzer^®^; category readings from both devices were averaged for further analyses.

Partial correlations adjusting for covariates of age, tobacco Q-score and FTND-M scores, which were found to significantly influence the correlation between piCO^+^ and iCO^TM^ Smokerlyzer^®^ readings, were performed using SPSS v25. Additionally, correlations between first and second piCO^+^ and iCO^TM^ Smokerlyzer^®^ readings and between the iCO^TM^ Smokerlyzer^®^ readings and FTND-M scores were also performed.

## RESULTS

The participants’ mean age was 47.9 years (Table 1). The average daily methadone dose was 69.7 mg, the mean tobacco Q-score was 12.3 cigarettes/day and the mean FTND-M score was 3.9. Most participants were male (92.5%), Malay (73.3%) and moderate smokers (55.5%).

**Table 1:** Baseline sociodemographic characteristics of study subjects (N=146 )

*Variable*	*Mean*	*SE*
Age (years)	47.89	0.85
Daily methadone dose (mg)	69.69	2.79
Tobacco Q-score (cigarettes/day)	12.25	0.67
Malay version of the Fagerström Test for Nicotine Dependence (FTND-M) score	3.94	0.17
	***n***	%
**Gender**		
Male	137	93.8
Female	9	6.2
**Race**		
Malay	107	73.3
Chinese	26	17.8
Indian	12	8.2
Other	1	0.7
**Tobacco Q-score category (cigarettes/day)**		
Light smoker (≤5)	28	19.2
Moderate smoker (6–19)	81	55.5
Heavy smoker (≥20)	37	25.3

**Figure 1:**
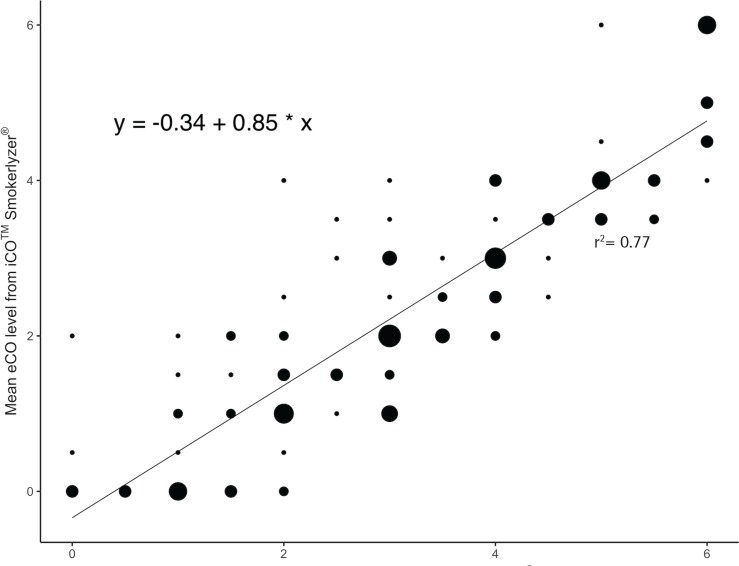
Weighted correlation (larger circles imply higher frequencies of individuals, and smaller circles imply lower frequencies of individuals) between exhaled carbon monoxide (eCO) level categories taken with the iCO^TM^ Smokerlyzer^®^ and the piCO^+^ Smokerlyzer^®^.

Mean eCO levels were significantly correlated between both devices (r=0.86, p<0.001; Figure 1), after adjusting for covariates of age, tobacco Q-score and FTND-M score. First and second device readings were significantly correlated with each other, after controlling for the same covariates (r=0.94 for categorical values of the iCO^TM^ Smokerlyzer^®^, p<0.001; r=0.91 for integer values of the piCO^+^ Smokerlyzer^®^, p<0.001; r=0.86 for categorical values of the piCO^+^ Smokerlyzer^®^, p<0.001). *Post hoc* analyses using the Bonferroni procedure revealed that iCO^TM^ Smokerlyzer^®^ readings were significantly lower than the corresponding piCO^+^ Smokerlyzer^®^ readings by 0.82 (95% CI: 0.69–0.94, p<0.001), and subsequent linear regression analyses confirmed a significant intercept of -0.34 (95% CI: -0.61 – -0.07, p=0.016). Mean eCO levels of the iCO^TM^ Smokerlyzer^®^ positively correlated with FTND-M scores (r=0.22, p<0.01).

## DISCUSSION

In this exploratory study, the significant correlation found between the readings of the iCO^TM^ Smokerlyzer^®^ and the piCO^+^ Smokerlyzer^®^, indicates that the iCO^TM^ Smokerlyzer^®^ may have validity equivalent to the piCO^+^ Smokerlyzer^®^ device that has been shown to be highly valid in discriminating between smokers and non-smokers^4-6^. With regard to reproducibility of the iCO^TM^ Smokerlyzer^®^, both readings were significantly correlated (r=0.94). Interestingly, the corresponding piCO^+^ Smokerlyzer^®^ value was lower (r=0.91 for raw values, r=0.86 when in grouped categories, p<0.001 for all three correlations), suggesting that the iCO^TM^ Smokerlyzer^®^ performed very well in terms of reproducibility when grouped into categories.

Additionally, the *post hoc* analysis finding and subsequent regression analyses with a significant intercept (Figure 1) suggest that one or more of the iCO^TM^ Smokerlyzer^®^ devices may be consistently yielding underestimates of eCO levels. Therefore, further guidelines are needed to recognize and rectify this calibration error, indicating that routine checks are needed against another calibrated device such as the piCO^+^ Smokerlyzer^®^. Finally, the finding that eCO levels of the iCO^TM^ Smokerlyzer^®^ correlated positively with the FTND-M scores warrants further research on whether reducing smoking in people with high nicotine dependence is a step towards smoking cessation.

This study did not assess raw values of the iCO^TM^ Smokerlyzer^®^ or abstinence cut-off points, thus restricting further analyses, such as Bland-Altman analysis, and the potential as a clinical utility. A study by Karelitz et al.^15^ had demonstrated lack of agreement and differences of 1.5–6.0 ppm between both monitors, thereby suggesting a lack of interchangeability between readings of both monitors. Additionally, the iCO^TM^ Smokerlyzer^®^ has a cited accuracy of 15% for each 1 ppm^7^, compared to <3% for the piCO^+^ Smokerlyzer^®3^. Consequently, the use of broad categories may indeed be supported, aiming to reduce the differences between devices, and in the process potentially increase interchangeability between readings of both monitors, which would need to be confirmed through further research.

Nevertheless, assessing the iCO^TM^ Smokerlyzer^®^ against the piCO^+^ Smokerlyzer^®^ has yielded some results of clinical significance. The piCO^+^ Smokerlyzer^®^ has been extensively studied and shown to be reproducible and reasonably accurate in determining patients who are abstinent, based on objective scores^4-6^. This study demonstrates that the categories obtained from the iCO^TM^ Smokerlyzer^®^ correlate with the categorical grouped integers shown on the piCO^+^ Smokerlyzer^®^. As such, there is a possibility for the more economical and user-friendly iCO^TM^ Smokerlyzer^®^ to be researched in greater detail in order to assess its suitability for use in research and clinical environments.

## CONCLUSIONS

The iCO^TM^ Smokerlyzer^®^ yielded highly reproducible results that are potentially comparable to the piCO^+^ Smokerlyzer^®^, pending further calibration guidelines. As a more economical and user-friendly device than the piCO^+^ Smokerlyzer^®^, the iCO^TM^ Smokerlyzer^®^ therefore has potential use in smoking cessation studies. Additionally, many individuals could benefit from using the iCO^TM^ Smokerlyzer^®^, to assess the true extent of their cigarette consumption, such that behavioral and pharmacological smoking interventions may be studied and implemented in a timely manner.
